# A Mini Review on the Contribution of the Anterior Cingulate Cortex in the Risk of Psychosis in 22q11.2 Deletion Syndrome

**DOI:** 10.3389/fpsyt.2018.00372

**Published:** 2018-08-17

**Authors:** Maria C. Padula, Elisa Scariati, Marie Schaer, Stephan Eliez

**Affiliations:** ^1^Friedrich Miescher Institute for Biomedical Research, Basel, Switzerland; ^2^Developmental Imaging and Psychopathology Laboratory, Office Médico-Pédagogique, Department of Psychiatry, University of Geneva, Geneva, Switzerland

**Keywords:** schizophrenia, saliency, DTI, resting-state fMRI, cortical thickness

## Abstract

22q11.2 deletion syndrome (22q11DS) is a neurogenetic disorder that causes a high risk of developing schizophrenia, thus representing a unique model for the investigation of biomarkers of psychosis. Cognitive and clinical risk factors have been identified as reliable predictors of schizophrenia in patients with 22q11DS and are currently used in the clinical practice. However, biomarkers based on neuroimaging are still lacking, mainly because of the analytic approaches adopted so far, which are almost uniquely based on the comparison of 22q11DS patients with healthy controls. Such comparisons do not take into account the heterogeneity within patients with 22q11DS, who indeed show various clinical manifestations. More recently, a number of studies compared measures of brain morphology and connectivity between patients with 22q11DS with different symptomatic profiles. The aim of this short review is to highlight the brain alterations found in patients with 22q11DS fulfilling ultra-high risk (UHR) criteria. Findings point to alterations in brain morphology and connectivity in frontal brain regions, and in particular in the anterior cingulate cortex, in patients with 22q11DS presenting UHR symptoms. These alterations may represent valuable biomarkers of psychosis in 22q11DS.

## Introduction

22q11.2 deletion syndrome (22q11DS) is considered the third most important risk factor for the development of schizophrenia after having a monozygotic twin (50%) or two affected parents (42%) (1, 2) Indeed, 30–40% of patients with 22q11DS develop psychosis by adulthood (2, 3). In addition, phenotypic characteristics of patients with 22q11DS affected by schizophrenia are similar to those of schizophrenic patients without the deletion (4, 5). Efforts are ongoing to identify predictive biomarkers, which could help us preventing the development of schizophrenia in patients with 22q11DS and that could be generalizable to idiopathic schizophrenia. A biomarker is defined as “*a characteristic that is objectively measured and evaluated as an indicator of normal biological processes, pathogenic processes, or pharmacological response”* (6). Therefore, alterations in cognitive processes or changes at the level of the brain could represent valuable biomarkers to predict the development of schizophrenia.

A number of investigations identified behavioral and clinical predictors of psychosis in 22q11DS (7–11). In a study pulling together data from two cohorts of patients with 22q11DS (Geneva and Tel Aviv) (7) we found that the presence of anxiety disorders and a lower baseline IQ predicted the development of schizophrenia. Another cross-site study conducted by the International Consortium on Brain and Behavior in 22q11 Deletion Syndrome (IBBC) included 829 22q11DS patients and showed that individuals with 22q11DS that develop psychosis have a steeper decline in verbal IQ (VIQ) from childhood to adulthood than controls and non-psychotic patients (11). Therefore, the cognitive decline represents another predictor of the development of psychosis in 22q11DS, in addition to the presence of anxiety disorders and a low IQ. We further showed that the Ultra High Risk (UHR) status and a decline in global functioning can also predict the conversion to psychosis in patients with 22q11DS (9). Our results showed that 27.5% of 22q11DS patients that meet the criteria for a UHR status convert to psychosis after 2.7 years, against the 4.5% conversion rate for non-UHR patients with the syndrome. Environmental factors also play a role in increasing the risk of psychosis in patients with 22q11DS. For instance, two recent investigations showed that preterm birth can be a risk factor of schizophrenia in 22q11DS (8, 10). In addition, it has been reported that the DNA methylation status differs in the patients with 22q11DS that develop mental disorders (12), thus suggesting a role of the environment and epigenetic factors in the risk of psychosis.

Despite the great number of studies showing alterations in brain morphology and connectivity in patients with 22q11DS compared to controls, there are still no valid biomarkers of psychosis based on neuroimaging. The findings concerning differences in brain morphology and connectivity in patients with 22q11DS are indeed very heterogeneous, thus preventing us to use them as biomarkers (13). Some studies investigated brain alterations associated to psychotic symptoms in 22q11DS in *post-hoc* analyses. For instance, in a recent review article, we concluded that higher symptoms severity is associated with altered long-range frontal connections (13). Fewer studies specifically investigated brain morphology and connectivity in 22q11DS patients with high psychotic symptoms compared to less symptomatic patients (14–21). The aim of this manuscript is to review evidence of specific alterations at the level of brain morphology and connectivity in patients with 22q11DS with a higher clinical risk to develop psychosis. In particular, we reviewed manuscripts that specifically investigated differences in subgroups of patients with 22q11DS that fulfill or not the criteria for UHR symptoms. We also propose that one specific region of the brain, the anterior cingulate cortex (ACC), is specifically impaired in the patients at high risk.

Several indicators confirm the involvement of the ACC in the risk of psychosis. This region of the brain is characterized by a great complexity from a structural and functional standpoint. Structurally, the ACC can be subdivided into into a rostral-ventral affective division and a dorsal cognitive division on the bases of its cytoarchitectonic and connectivity patterns (22, 24). Functionally, the ACC is part of different brain networks and is important for emotion regulation and cognitive processes. In particular, two main functions may relate the ACC to a greater risk of psychosis: its involvement in self-monitoring and its role in salience processing, which have both been associated with the psychopathology of schizophrenia. Self-monitoring is defined as the ability to correctly attribute the origin of internally generated stimuli. Alterations in self-monitoring have been reported in patients with schizophrenia (23). For instance, impaired attribution of self-generated thoughts can cause the manifestation of hallucinations, and the ACC has been involved in this misattribution of internal speech (24, 25). As part of the salience network, the ACC is also important in saliency attribution to internal and external stimuli. Impaired salience attribution may in turn be responsible for the manifestation of hallucinations and delusions (26).

## Brain morphology in patients with 22q11DS at high risk of psychosis

To the best of our knowledge, only four studies to date compared measures of brain morphology between patients with 22q11DS without UHR symptoms or psychosis and patients with 22q11DS with a diagnosis of UHR or psychosis [(16, 27–29), Table 1]. In Dufour et al. (27), we compared the volume of the ACC between controls and patients with 22q11DS, as well as between psychotic and non-psychotic patients with the syndrome (27). Reduced ACC volume was evident in patients with 22q1DS with higher psychotic symptoms. Further studies from ours and other cohorts investigating the developmental trajectories of cortical thickness, consistently found accelerated cortical thinning of frontal brain regions in 22q11DS patients with higher symptoms compared to less symptomatic patients with the syndrome (28, 29). This was confirmed by our recent investigation (16) in which we observed accelerated cortical thinning of frontal cortex and ACC in patients with 22q11DS that meet the criteria for a UHR status compared to non-UHR patients.

**Table 1:** Brain morphology and connectivity in patients with 22q11DS at high risk of psychosis.

**References**		**Cohort**	**Sample size**	**Measure**	**Results**
**BRAIN MORPHOLOGY**					
Padula et al. (16)		Geneva	22 psy+, 22 psy–	Cortical thickness	Bilateral superior frontal cortex, right prefrontal cortex, left anterior cingulate cortex*, left inferior parietal cortex.
Dufour et al. (27)		Geneva	24 psy+, 18 psy–	Gray matter volume	Cingulate gyrus * (right dorsal anterior cingulate cortex and cingulate body).
Ramanathan et al. (28)		SUNY Upstate Medical University	18 psy+, 57 psy–	Cortical thickness	Frontal lobe, superior frontal cortex.
Schaer et al. (29)		Geneva	6 psy+, 13 psy–	Cortical thickness	Right fusiform/lingual region, left superior frontal gyrus.
**STRUCTURAL CONNECTIVITY**					
Kikinis et al. (14)		SUNY Upstate Medical University	9 psy+, 41 psy–	Fractional anisotropy, axial diffusivity, radial diffusivity, mean diffusivity	Right superior longitudinal fasciculus, corpus callosum, right superior corona radiata, right internal capsule.
Padula et al. (17)		Geneva	31 psy+, 31 psy–	Fractional anisotropy, axial diffusivity, radial diffusivity, tractography, graph theory	Right amygdala, left posterior cingulate cortex, left parahippocampal cortex, right anterior cingulate cortex*, inferior longitudinal fasciculus, cingulate gyrus*.
Roalf et al. (18)		University of Pennsylvania and Children's Hospital of Philadelphia	27 psy+, 12 psy–	Fractional anisotropy, axial diffusivity, radial diffusivity, mean diffusivity	Cingulate bundle*, uncinate fasciculus.
Sandini et al. (19)		Geneva	31 psy+, 31 psy–	Covariance of cortical thickness	Superior frontal gyrus, anterior and middle cingulate gyri*.
**FUNCTIONAL CONNECTIVITY**					
Schreiner et al. (20)		University of California, Los Angeles, SUNY Upstate Medical University	18 psy+, 38 psy–	Functional connectivity	Anterior cingulate cortex/precuneus*, default mode network*, left executive network, salience network*.
Zöller et al. (21)		Geneva	28 psy+, 29 psy–	Variability of resting-state blood oxygenated level- dependent signal	Dorsal anterior cingulate cortex*, prefrontal cortex, orbitofrontal cortex, V2.
Scariati et al. (30)		Geneva	13 psy+, 17 psy–	Functional connectivity	Anterior cingulate cortex*, inferior frontal gyrus (pars triangularis), precentral gyrus, rectus gyrus, superior parietal gyrus.

*Indicates findings involving the anterior cingulate cortex. Note that the study cohorts recruited in the same geographic location or by the same research group are not independent. For instance, the patients and controls recruited in Geneva (16, 17, 19, 21, 27, 29, 30) overlap across the different studies. The same holds true for the other cohorts being recruited in the other different geographic areas. The data collected across the different locations are instead independent. Psy+, patients with moderate to severe ultra-high risk symptoms; Psy–, patients with low ultra-high risk symptoms.

## Structural connectivity in patients with 22q11DS at high risk of psychosis

Few studies investigated white matter connectivity in patients with 22q11DS with and without UHR symptoms [(14, 17, 18), Table 1]. Initial investigations used measures of white matter microstructural integrity, namely fractional anisotropy (FA), axial (AD), and radial diffusivity (RD) extracted from specific white matter pathways (14, 18). In a cohort of 22q11DS patients recruited from the University of Pennsylvania and the children hospital of Philadelphia Roalf and colleagues showed that individuals with 22q11DS with higher prodromal symptoms have a lower mean FA in the cingulate gyrus (CG), thus suggesting altered white matter integrity of this white matter bundle in association to a greater risk of psychosis. This was confirmed by our recent investigation (17) in which we showed that reduced FA and increased RD in the CG significantly discriminated patients with 22q11DS with higher and lower symptoms severities. In addition to diffusion measures, we investigated structural network integrity using tractography and measures of graph theory (17). We found that a pattern of disconnectivity of the ACC discriminated 22q11DS patients with UHR symptoms.

Structural covariance of cortical thickness represents another method to investigate structural connectivity between brain regions. Here, structural connectivity is measured as the correlation between thickness values extracted from regions of interest. Indeed, it is assumed that morphology is correlated between regions that are structurally connected, due to the mutually trophic effect of axonal connections. We found that patients with 22q11DS with high psychotic symptoms are characterized by altered structural covariance of the anterior cingulate and medial prefrontal cortices, which supports the findings based on white matter connectivity (19).

## Functional connectivity in patients with 22q11DS at high risk of psychosis

Findings in functional connectivity measured with resting-state fMRI further support the involvement of the ACC in the risk of psychosis in patients with 22q11DS (Table 1). A large multicentric investigation including 22q11DS individuals assessed at the University of California Los Angeles (UCLA) and at the SUNY Upstate Medical University in Syracuse (20) used a multivariate approach to identify a pattern of brain alterations that discriminated patients with 22q11DS according to different symptomatic profiles. More specifically, a classifier was trained using network connectivity data from patients with 22q11DS collected at the UCLA. The classifier was then tested in the SUNY cohort in order to provide a generalizable classification. When discriminating patients with 22q11DS with high and low symptoms severities, the combination of four networks gave a prediction accuracy of 78%: the ACC/precuneus network, the default mode network (DMN), the central executive network (CEN), and the saliency network (SN). Three out of these networks include the ACC, thus confirming how alterations in this functionally complex brain region can compromise networks integrity and result in the manifestation of psychotic symptoms. These findings are further supported by our recent investigations, in which we showed disconnectivity of the ACC (30) and altered BOLD (Blood Oxygenated Level Dependent) signal variability (21) in patients with 22q11DS with higher UHR symptoms.

## Discussion

The results of the studies reviewed in this manuscript point to specific brain alterations characteristic for a higher risk of psychosis in patients with 22q11DS. Even though some limitations can be identified (lack of independency between study cohorts, moderate sample sizes, and sometimes heterogenous findings) few brain regions and connections emerge as being associated to a greater risk to develop psychosis. In particular, one consistent finding across studies is an involvement of the ACC. These results are in line with findings reported in individuals at high risk without the 22q11.2 deletion (31–38). Altered morphology of the ACC has indeed been reported in patients at ultra-high risk (UHR) without 22q11DS (31, 32). In addition, alterations in the ACC are present and worsen in at risk individuals that convert to psychosis (32–36) and are found in non-deleted patients with schizophrenia (37, 38). These findings therefore confirm the role of the ACC in the development of psychosis. Of note, other regions recurrently reported to be altered in individuals at risk without 22q11DS are the frontal cortex and the insula (33, 34), in line with the findings in individuals with 22q11DS fulfilling UHR criteria.

Several neuropathological mechanisms may explain the altered activity and connectivity of the ACC. A widely accepted hypothesis suggests the impairment of parvalbuminergic (PV) interneurons. These fast-spiking cells significantly influence the activity of pyramidal neurons and are involved in gamma and theta oscillations necessary for the short- and long-range communication between brain regions. Therefore, impairments in PV interneurons may explain the alterations in ACC connectivity found in patients with schizophrenia and 22q11DS. Reduced density of PV interneurons has been found in postmortem brains of patients with schizophrenia (39), mainly in the frontal cortex, as well as in mouse models of schizophrenia (40), thus confirming this hypothesis. Another complementary theory proposed a reduced activation of the Glutamate NMDR receptor, which has been associated to increased oxidative stress (41). The altered redox balance may in turn be responsible for the reduction in PV interneurons (34).

A more recent hypothesis put forward the involvement of a particular type of neurones, the so called “*Von Economo neurons,”* which are specifically located in the ACC and fronto-insular cortex. Von Economo neurons have been shown to be involved in long-range transmission and develop during the first years of age, thus suggesting their role in high order functional domains such as emotion regulation, motor control, decision making and social cognition (42). Furthermore, they contain receptors for neurotransmitters, such as vasopressin, dopamine, and serotonin, involved in complex behaviors. Interestingly, it has been shown that the density of Von Economo neurons in the ACC is associated with the age at onset and the duration of illness in patients with schizophrenia (40, 42) thus suggesting that the pathology of these neurons may explain the ACC structural and functional impairments observed in patients at risk of psychosis.

The fact that one specific brain region, the ACC, may be involved in the risk of schizophrenia in patients with 22q11DS opens the important perspective of targeting this region for treatment. Indeed, non-invasive brain stimulation approaches such as neurofeedback, transcranial magnetic stimulation, and transcranial electrical stimulation may be used to modulate the activity of the ACC and restore the function of networks where the ACC takes part. However, the realization of such non-invasive brain stimulation approaches is still challenging, especially because the ACC is a deep region in the brain and thus difficult to target. In the future, further investigations are needed with larger samples and cross-site cohorts in order to confirm the role of the ACC in the risk of psychosis in these patients. However, the findings obtained so far give interesting directions for future research in the field, thus allowing to narrow down the search for biomarkers of psychosis in 22q11DS.

## Author contributions

MP wrote the manuscript. All the authors contributed to the conception of the manuscript and the interpretation of the literature and critically revised the manuscript.

### Conflict of interest statement

The authors declare that the research was conducted in the absence of any commercial or financial relationships that could be construed as a potential conflict of interest.
